# Impact of pension income on healthcare utilization of older adults in rural China

**DOI:** 10.1186/s12939-023-01985-5

**Published:** 2023-08-26

**Authors:** Peizhe Yan, Fenghang Li, Stephen Nicholas, Elizabeth Maitland, Jialong Tan, Chen Chen, Jian Wang

**Affiliations:** 1 Dong Fureng Institute of Economic and Social Development, Wuhan University, Wuhan, Hubei Province, China; 2 Australian National Institute of Management and Commerce, Australian Technology Park, Sydney, NSW Australia; 3 Newcastle Business School, University of Newcastle, Newcastle, NSW Australia; 4 School of Management, University of Liverpool, Liverpool, England; 5 Department of Global Health, School of Public Health, Wuhan University, Hubei Province, 115 Donghu Road, Wuhan, 430079 China; 6 Center for Health Economics and Management at the School of Economics and Management, Wuhan University, Hubei Province, Room A201, Wuhan, 430079 China

**Keywords:** New Rural Pension Scheme, Income shock, Healthcare utilization, Rural residents, Over-the-counter drug

## Abstract

**Objective:**

In China, rural residents experience poorer health conditions and a higher disease burden compared to urban residents but have lower healthcare services utilization. Rather than an insurance focus on enhanced healthcare services utilization, we aim to examine that whether an income shock, in the form of China’s New Rural Pension Scheme (NRPS), will affect outpatient, inpatient and discretionary over-the-counter drug utilization by over 60-year-old rural NRPS residents.

**Methods:**

Providing a monthly pension of around RMB88 (USD12.97), NRPS covered all rural residents over 60 years old. Fuzzy regression discontinuity design (FRDD) was employed to explore the NRPS causal effect on healthcare services utilization, measured by outpatient and inpatient visits and discretionary over-the-counter drug purchases. The nationwide China Health and Retirement Longitudinal Study (CHARLS) 2018 provided the data.

**Results:**

Without significant changes in health status and medication needs, 60-plus-year-old NRPS recipients significantly increased the probability of discretionary OTC drug purchases by 33 percentage points. NRPS had no significant effect on the utilization of outpatient and inpatient utilization. The increase in the probability of discretionary OTC drug purchases from the NRPS income shock was concentrated in healthier and low-income rural residents. Robustness tests confirmed that FRDD was a robust estimation method and our results are robust.

**Conclusion:**

NRPS was an exogenous income shock that significantly increased the probability of discretionary over-the-counter drug purchases among over 60-year-old rural residents, but not the utilization of inpatient or outpatient healthcare services. Income remains an important constraint for rural residents to improve their health. We recommend policymakers consider including commonly used over-the-counter drugs in basic health insurance reimbursements for rural residents; provide health advice for rural residents to make discretionary over-the-counter drug purchases; and to mount an information campaign on over-the-counter drug purchasing in order to increase the health awareness of rural residents.

## Introduction

Income-related socioeconomic factors have a significant impact on the health of a population [1, 2], with the healthy aging of those in the lower socioeconomic strata emerging as a major global challenge. Rural populations have both lower income and poorer health. In China and the U.S., the health status of rural residents is poorer than urban residents [3, 4]. Many studies have demonstrated that rural residents do not exhibit a strong awareness or behavior to maintain or improve their health, even when they already have chronic conditions [5, 6]. Chinese rural residents have fewer sources of health information than urban residents, and significantly poorer health literacy [7]. Besides weaker health awareness, Chinese rural residents have poorer innate endowments in medical resource accessibility and per capita healthcare resources [8]. An empirical analysis using the China Health and Nutrition Survey (CHNS) found that the ratio of the actual urban–rural healthcare utilization was 1.744 [9], identifying the urban–rural income gap as an important cause of the unequal rural–urban access to healthcare.

Most of the existing literature focuses on the impact of health insurance on healthcare resource utilization. Regarding rural residents in China, the most studied is the New Rural Cooperative Medical System. But there is evidence that the implementation of the New Rural Cooperative Medical System has not successfully reduced the out-of-pocket costs of rural residents [10–12]. The literature on health insurance has not reached uniform conclusions, and cannot fully explain changes, in the use of health care resources among rural residents [13, 14]. Rather than an insurance focus on medical utilization, there is emerging research on the impact of pension receipts on healthcare utilization in developing countries [15], and poverty alleviation strategies on non-free healthcare services in China [16].

Self-medication behaviors using over-the-counter (OTC) drugs are prevalent in China [17]. More than half of the Chinese over the age of 45 self-medicate, and the prevalence of self-medication among the Chinese elderly is increasing [18]. Most of these self-medications are delivered via over-the-counter drugs [19]. This persistent urban–rural disparity in OTC drug use might suggest that rural residents have less access to professional healthcare resources [20]. In the absence of professional family doctors and the distance from hospitals, OTC drugs seem to be the most accessible healthcare resource for rural residents compared to urban residents. Compared to urban respondents, rural residents were more likely to consider drug price as a decision factor in purchasing over-the-counter drugs [17]. Proper self-medication can reduce the long-term healthcare burden at the individual and societal level [21, 22]. In addition to conventional healthcare resources such as inpatient and outpatient care, self-medication, especially over-the-counter drugs, deserves special attention for rural residents.

Taking this new track, as our first contribution, the purpose of our research is to investigate whether an income shock in the form of China’s New Rural Pension Scheme (NRPS) impacted the healthcare services utilization of Chinese rural residents who are 60 years old and older. Healthcare services in our paper comprise outpatient services, inpatient services and discretionary OTC drug purchases. The existing literature mainly focuses on inpatient and outpatient services [23, 24].

Our second contribution is to assess the impact of NRPS on discretionary OTC drug purchases as well as the conventional inpatient and outpatient service measures of healthcare services utilization. OTC drug purchases are a non-neglectable part of the monthly medical expenditure for the older population. Discretionary OTC drug purchase deserves special attention because it is the most accessible and affordable health input and the typical healthcare services health behavior by rural residents. The rational use of over-the-counter drugs can relieve once-off illnesses or symptoms, reducing disease harm effects, and is a form of self-management healthcare, reflecting a long-term health capital investment to avoid disease [25–28].

Rural residents consume health services as a consumption good when they are ill and as a capital investment in their future health [29, 30]. We focus on the exogenous income shock effect on medical service purchases brought by the implementation of the NRPS. NRPS raises rural residents’ income, which will allow them to consume more, including preventative healthcare capital investments and therapeutic healthcare, or to save. NRPS provides all 60-year-olds with a fixed amount of regular income in the long term [31, 32]. To measure healthcare services utilization, we focus on discretionary OTC drug purchases, inpatient services and outpatient services.

In the late 1990s, Chinese authorities launched several pilot projects to better improve old age protection for rural residents, aiming to establish an old-age insurance scheme for rural areas (also known as the Old Rural Pension Scheme, ORPS). Given the low awareness of insurance among rural residents and the fact that the main source of funding for ORPS is from individual contributions, the ORPS pilot did not gain the support of rural residents.

To cope with a rapidly aging population, China launched the New Rural Pension Scheme (NRPS) in 2009, and by 2012, all 2,853 counties or areas in China were covered, with 460 million people insured. Rural residents who are 16 years old or older (excluding students) and rural workers who are not participating in the basic pension insurance can enroll in the NRPS at their household registration locations voluntarily. NRPS uses a combination of individual contributions, government subsidies and collective subsidies for financing. Compared with the old rural insurance scheme, which relied mainly on rural residents’ contributions, NRPS emphasizes the government's protection function. Specifically, there are five levels of NRPS premium, ranging from RMB100 (USD14.74) to RMB500 (USD73.70) per year, within which rural residents can voluntarily choose their premium levels, while local governments can also set additional contribution levels and additional subsidies. The NRPS has also improved the pension payment structure, with the pension account consisting of two parts: the basic pension and the individual account. After meeting the NRPS requirements, residents under the age of 60 can receive not only the basic pension, which is covered by the government, but also the individual account pension. Participants who pay a higher premium level will receive a larger pension.

NRPS stipulated that all enrolled NRPS members (regardless their sex) received the pension at age of 60. Rural residents who were already 60 years old at the time of the implementation of NRPS received the basic pension, although they paid no premiums, provided their children were enrolled in the NPRS. For most regions, the minimum monthly basic pension was RMB1056 (USD155.65) per year [33], which is a modest supplement to the average rural residents’ 2018 RMB14617 (USD2154.55) yearly income [34].

The exact timing of when rural residents receive their pensions will be affected by the seasonality and intensity of policy implementation at the county level. For example, some local governments centralize pension payments at the end of the year, and there will be early and or delayed payments [31]. As a result, some participants who reached the age of 60 did not receive their pensions when reaching aged 60, while a small number of participants under the age of 60 received their pensions.

Pensions as additional income can relax budget constraints, allowing the consumption of additional healthcare resources. The healthcare resources we focus on include inpatient, outpatient and over-the-counter drugs. The amount of pension is small and the price elasticity of demand is more elastic for drugs than outpatient and inpatient services. Therefore, we propose two assumptions: First, pension receipt increases the probability of discretionary purchase of over-the-counter drugs among respondents, and second, pension receipt may not increase respondents' utilization of inpatient or outpatient resources.

## Methods

We use regression discontinuity design (RDD) to examine whether an income shock, in the form of China’s New Rural Pension Scheme (NRPS), will affect healthcare utilization by over 60-year-old rural NRPS residents. Output variables include outpatient, inpatient and discretionary over-the-counter drug utilization. We conduct sub-sample analyses based on respondents' health status and income. Continuity tests, data heaping tests, placebo tests are performed and different bandwidths are applied to check the robustness of our results.

### RDD Model

**Fig. 1:**
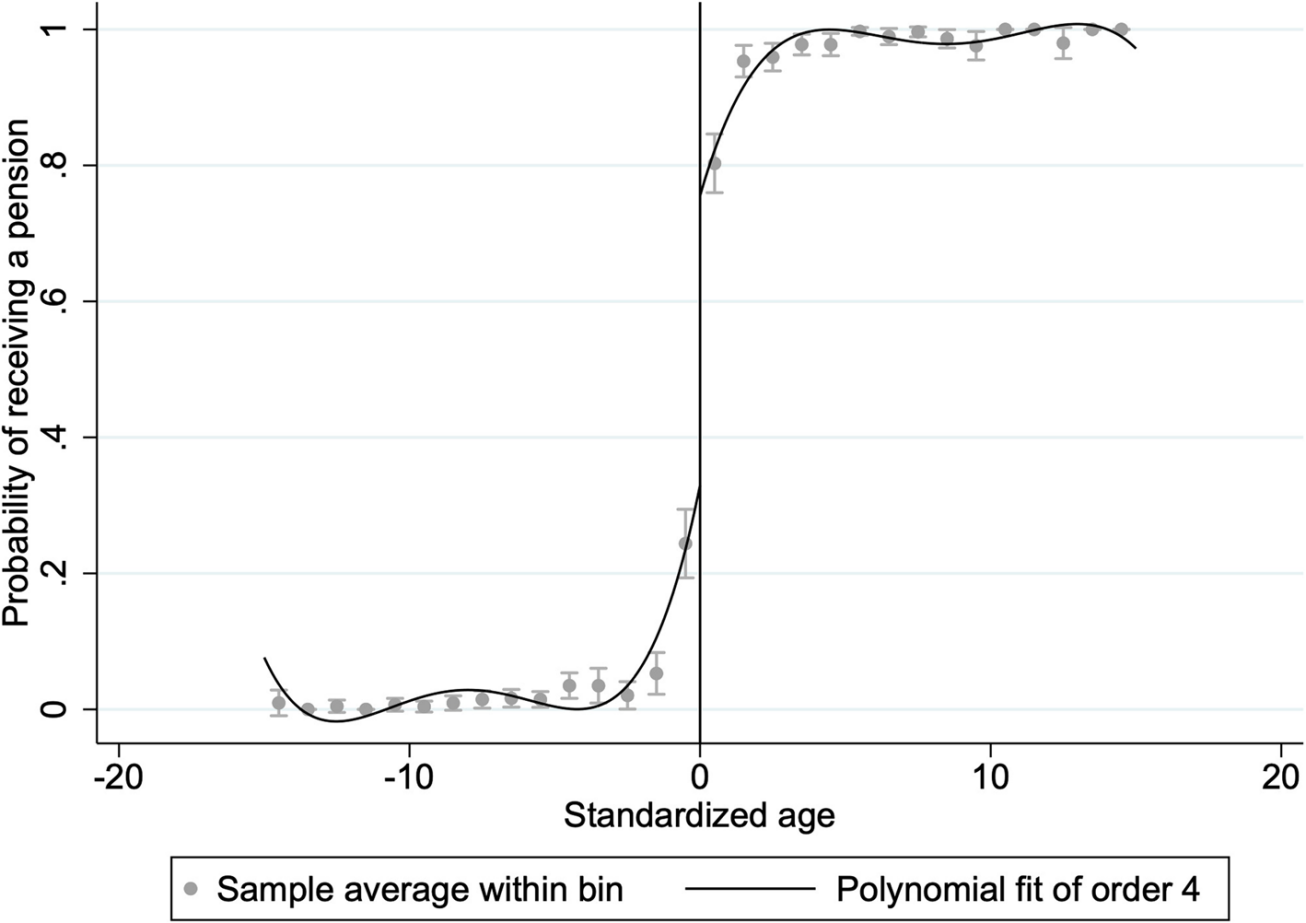
Discontinuity in the probability of receiving pensions

To test healthcare utilization by NRPS residents, we use a regression discontinuity design (RDD) [35]. This method requires the probability of receiving a pension for rural residents participating in NRPS having a distinct discontinuity at age 60. Figure 1 shows that a significant discontinuity exists at age 60 by conducting a preliminary test for age discontinuity, which allows us to use the regression discontinuity design. Regression discontinuity design (RDD) can be classified as sharp regression discontinuity design (SRDD) and fuzzy regression discontinuity (FRDD). FRDD allows for a small jump in the probability of an individual being assigned to the experimental group at the discontinuity, spanning between zero and one. It means that the individuals on either side of the discontinuity do not coincide with the actual intervention status received, such that individuals on the right side of the discontinuity do not necessarily receive the intervention, and individuals on the left side of the discontinuity may also receive the intervention. The actual age of receiving the pension may not always be exactly 60 years old given the differences in policy and financial status across regions and the seasonality and intensity of policy implementation at the county level [31]. FRDD is thus more germane to the bulk of clinical and policy questions in the sphere of healthcare [36]. Therefore, the discontinuity point formed by the retirement system is a fuzzy discontinuity and FRD is used in our study.

Using age 60 as the cutoff, a nonparametric approach was used to estimate the FRDD instrumental variables [37]. Medical resource utilization, for individual i can be expressed as:

1 $${Y}_{i}={Y}_{1i}{D}_{i}+{Y}_{0i}(1-{D}_{i})={Y}_{0i}+({Y}_{1i}-{Y}_{0i}){D}_{i}$$

where ${D}_{i}$ represents the pension-receiving status of an individual, ${D}_{i}$=1 represents the individual receiving the pension and ${D}_{i}$=0 represents the individual who does not receive the pension. ${Y}_{i}$ is outcome variable and represents the medical resource utilization for individual *i*; ${Y}_{1i}$ represents the potential outcome of medical resource utilization when the individual receives a pension and *Y*_*0i*_ represents the potential outcome of medical resource utilization when the individual does not receive a pension.

The causal effect of pensions on individual outcome variables is:

2 $$\tau ={Y}_{i1}-{Y}_{i0}=E[{Y}_{1}|X]-E[{Y}_{0}|X]$$

Near the cutoff, the simplicial equation expressed as a conditional expectation function of Y_i_ is:

3 $${E[Y}_{i}\left|{x}_{i}\in \left[{c}_{0}, {c}_{0}+\Delta \right]-{E[Y}_{i}\right|{x}_{i}\in \left[{c}_{0}-\Delta , {c}_{0}\right]\approx \tau \pi$$

Processing variable D_i_ in the first stage:

4 $${E[Y}_{i}\left|{D}_{i}\in \left[{c}_{0}, {c}_{0}+\Delta \right]-{E[Y}_{i}\right|{D}_{i}\in \left[{c}_{0}-\Delta , {c}_{0}\right]\approx \pi$$

Therefore, the local average treatment effect (LATE) of the regression discontinuity of pensions on medical resource utilization is estimated by the parsimonious formula after finding the left and right limits at the discontinuity for Eq. (1) as:

5 $$\tau =\frac{\underset{x\downarrow c}{lim}E\left[{Y}_{i}|{x}_{i}\in \left[{c}_{0}, {c}_{0}+\Delta \right]\right]-\underset{x\uparrow c}{lim}E\left[{Y}_{i}|{c}_{0}-\Delta , {c}_{0}\right]}{\underset{x\downarrow c}{lim}E\left[{D}_{i}|{x}_{i}\in \left[{c}_{0}, {c}_{0}+\Delta \right]\right]-\underset{x\uparrow c}{lim}E\left[{D}_{i}|{c}_{0}-\Delta , {c}_{0}\right]}=\frac{\underset{x\downarrow c}{lim}E\left[Y|X\right]-\underset{x\uparrow c}{lim}E\left[Y|X\right]}{\underset{x\downarrow c}{lim}E\left[D|X\right]-\underset{x\uparrow c}{lim}E\left[D|X\right]}$$

X is denoted as a set of predetermined variables.

In terms of the specific operations of the nonparametric approach, this paper draws on Calonico’s method [37] to obtain the numerator and denominator of the estimated Eq. (5).

How to choose the appropriate bandwidth is a key issue in performing nonparametric estimation. The (smaller) larger the bandwidth chosen, the (smaller) larger the sample size included, and the representativeness will (decrease) improve, but the reliability will (improve) decline. The optimal bandwidth for the baseline regression was selected by using the minimum mean square error to balance representativeness and credibility [38]. All analyses are performed by Stata16.0.

### Database and sample selection

We used cross-sectional data from the newly released 2018 China Health and Retirement Longitudinal Study (CHARLS). CHARLS data after 2018 is not available to the public. A representative sample covering economic, health, medical and retirement aspects of households and individuals, the CHARLS database has over 19,816 observations on over 45-year-old rural residents from 150 county-level and 450 village-level units across China [39]. In our analysis, we include NRPS enrollees who were 45–75 years old and whose household registration was rural, with no missing data on the outcome variables, which yielded 7878 observations.

### Dependent variables

We used three core healthcare services utilization categorical explanatory variables: 1) whether they made discretionary OTC drug purchases assessed by the question "Did you purchase medicine during the past month (not including prescription medications)"; 2) whether they visited an outpatient clinic during the past month; 3) and whether they received inpatient care in the past year.

### Covariates

Covariates include socio-demographic variables and health-related variables.

Socio-demographic variables include sex, marital status, income and education level. Income is calculated as personal wage income plus net agricultural income minus pensions, where net agricultural income is the value of the current year's crop minus the proportion for own use, minus input costs. Since a significant proportion (30%) of income was negative and therefore cannot be treated logarithmically, the quartile method was adopted. To accurately identify eligibility for retirement, we generate monthly level age data. For example, the monthly age of a rural resident born in January 1968 is 50 plus one-twelfth, which is 50.083.

Health-related variables comprised self-rated health (0 for bad and very bad answers and 1 otherwise); the number of chronic diseases; whether they took a prescribed drug; health insurance (1 yes and 0 no); and depression status. Prescribed drug is an integrated variable consisting of multiple questionnaire responses. In the CHARLS 2018 questionnaire, for certain health conditions (such as diabetes and hypertension) the question was "whether you take the following measures to treat a certain disease" which included both Chinese and Western medicine. In addition to chronic diseases, for certain ailments (such as pain), the respondents were also asked whether they had taken any prescribed treatment measures, including both Chinese and Western medicine. Individuals were assigned a value of 1 when they took prescribed drugs and 0 otherwise. Based on the Center for Epidemiologic Studies Depression Scale (CES-D-10) a score of 10 and above in CHARLS was used to define 1 depression, 0 otherwise [40].

## Results

### General characteristics of the respondents

Table 1 presents the general characteristics of the respondents, including outcome variables and convertibles. In addition to the general characteristics of the overall population, Table 1 also show the characteristics of the different age groups, using 60 as a cut-off.

**Table 1:** General characteristics of the respondents

	All (*n* = 7878)	Age ≤ 60 (*n* = 3,992)	Age > 60 (*n* = 3,886)
**Discretionary OTC drug purchase (Mean, SD)**	0.592 (0.491)	0.565 (0.495)	0.619 (0.485)
**Outpatient (Mean, SD)**	0.165 (0.371)	0.169 (0.375)	0.161 (0.368)
**Inpatient (Mean, SD)**	0.156 (0.363)	0.122 (0.328)	0.191 (0.393)
**Sex (Mean, SD)**	0.460 (0.498)	0.453 (0.497)	0.468 (0.499)
**Marital status (n, %)**			
*Married with spouse present*	6,383 (81.02)	3,319 (83.14)	3,064 (78.85)
*Married but not living with spouse temporarily for reasons such as work*	609 (7.73)	442 (11.07)	167 (4.30)
*Separated*	25 (0.32)	17 (0.43)	8 (0.21)
*Divorced*	60 (0.76)	36 (0.90)	24 (0.62)
*Widowed*	761 (9.66)	162 (4.06)	599 (15.41)
*Never married*	40 (0.51)	16 (0.40)	24 (0.62)
**Education (n, %)**			
*Illiterate*	1,950 (24.75)	619 (15.51)	1,331 (34.25)
*Did not Finish Primary School*	1,938 (24.60)	874 (21.89)	1,064 (27.38)
*Sishu/Home School*	2 (0.03)	0 (0)	2 (0.05)
*Elementary School*	1,844 (23.41)	1,011 (25.33)	833 (21.44)
*Middle School*	1,659 (21.06)	1,162 (29.11)	497 (12.79)
*High School*	449 (5.70)	306 (7.67)	143 (3.68)
*Vocational School*	27 (0.34)	15 (0.38)	12 (0.31)
*Two-/Three-Year College/Associate degree*	8(0.10)	5 (0.13)	3 (0.08)
*Four-Year College/Bachelor’s degree*	1 (0.01)	0 (0)	1(0.03)
**Income (net of pension) (Mean, SD)**	2.475 (1.117)	2.650 (1.169)	2.295 (1.029)
**Self-rated health (Mean, SD)**	0.672 (0.462)	0.727 (0.445)	0.616 (0.482)
**Number of chronic diseases** **(Mean, SD)**	0.691 (1.011)	0.623 (0.962)	0.761 (1.055)
**Prescribed drugs (Mean, SD)**	0.470 (0.499)	0.448 (0.497)	0.493 (0.500)
**Medical Insurance (Mean, SD)**	0.981 (0.136)	0.985 (0.120)	0.976(0.150)
**Depression (Mean, SD)**	0.400 (0.490)	0.385 (0.486)	0.416(0.493)
**Total Observations**	7,878	3,992	3,886

*Abbreviations:* *SD* Standard deviation

### Impact of NRPS on the utilization of healthcare services

Table 2 shows the estimation results for outpatient and inpatient medical service utilization and discretionary over-the-counter drug utilization. The first stage results are about the relationship between age and access to pensions. The standardized age at the discontinuity point is 60 years old. From Fig. 1, the probability of receiving an NRPS pension jumped significantly around age 60, which is consistent with the design of the NRPS system. In addition, the first stage results in Table 2 show the effect of age on pension receipt, and the results are all significant, which provides ample evidence that the cutoff at age of 60 years old was appropriate as an "instrumental variable" for whether to receive a pension.

**Table 2:** Regression result

	Panel A: Inpatient		Panel B: Outpatient		Panel C: Discretionary OTC drug purchase	
	First stage	Inpatient	First stage	Outpatient	First stage	Discretionary OTC drug purchase
Pension	0.420***	0.099	0.358***	0.110	0.303***	0.329*
	0.043	0.080	0.048	0.109	0.054	0.188
95% CI	[0.334,0.504]	[-0.058,0.255]	[0.262,0.453]	[-0.104,0.324]	[0.196,0.409]	[-0.040,0.698]
Bandwidth		3.379		2.739		2.299
Sample size		740; 1187		645; 891		556; 763

Robust criteria errors for clustering to the individual level are reported in parentheses, *p < 0.1, ***p < 0.01. The default minimum mean square error bandwidth is used in the table

**Fig. 2:**
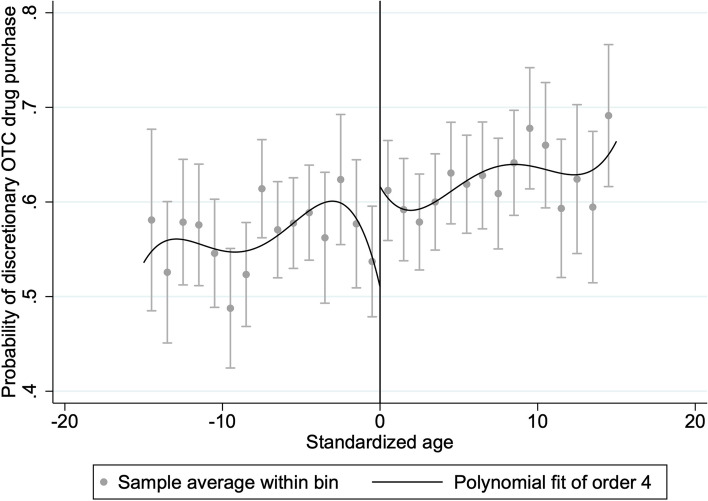
The relationship between discretionary OTC drug purchase and NRPS

Following Calonico’s method [38], we calculated the optimal bandwidth under the minimum mean square error and estimated the regression results for the fuzzy discontinuity. From Table 2 Panel A, when the dependent variable is inpatient care, the optimal bandwidth is 3.379 years. The treatment effect of discontinuity regression is not significant with the growth of 9.9 percentage point. From Table 2 Panel B, the optimal bandwidth is 2.739 years when the dependent variable is outpatient visits and the treatment effect of discontinuity regression is not significant with the growth of 11 percentage point. In Table 2 Panel C, when the dependent variable is discretionary OTC drug purchases, the optimal bandwidth is 2.299 years and the discontinuity regression treatment effect is significantly positive. These regression results show that NRPS pension had no significant effect on inpatient and outpatient healthcare services utilization, but had a significant effect on discretionary OTC drug purchasing behavior. Figure 2 visualizes a significant upward jump in the probability of discretionary OTC drug purchasing behavior of individuals after receiving an NPRS pension, where the pension income shock increased the probability of discretionary OTC drug purchases by 33 percentage points among rural residents. When rural residents receive the pension, their income increase one fixed amount compared with no-pension status. The results show that pension receipt significantly increases the probability of spontaneous drug purchase by rural residents. The effect of pensions on outpatient and inpatient services was also positive, but not significant.

### Subsample analysis

We conducted a subsample analysis based on health status and income. Health status includes respondents' self-rated health status, whether they are depressed and whether they have chronic diseases. Based on their median household wage income plus net agricultural income, respondents were divided into high-income and low-income groups. In the subsample analysis, we found there were no significant changes in outpatient and inpatient service utilization at age 60. But we confirmed a significant increase in the probability of discretionary OTC drug purchases from the NRPS income shock in healthier populations. Table 3 shows that the impact of the pension income shock on over-the-counter drug purchases was more pronounced among those who were free of depression and chronic illness and who self-rated their health status as moderate or healthy. This suggests that healthier individuals were more likely to increase their investment in their health through over-the-counter discretionary OTC drug purchases after receiving NRPS compared to unhealthy individuals.

**Table 3:** Subgroup analysis

		Drug purchase	95% CI	Bandwidth (Sample size)	Inpatient	95% CI	Bandwidth (Sample size)	Outpatient	95% CI	Bandwidth (Sample size)
Depression	Not depression	0.276*	[-0.050, 0.602]	3.227	0.060	[-0.135, 0.257]	3.367	0.243	[-0.190, 0.676]	2.210
		(0.166)		(422; 649)	(0.100)		(432; 681)	(0.221)		(317; 434)
	Depression	0.182	[-0.123, 0.488]	3.254	0.145	[-0.080, 0.370]	3.789	0.034	[-0.224, 0.293]	3.248
		(0.156)		(301; 490)	(0.115)		(341; 565)	(0.132)		(296; 470)
Self-rated Health	Moderate or Healthy	0.270*	[-0.027, 0.567]	3.029	0.024	[-0.114, 0.163]	3.675	0.149	[-0.098, 0.397]	2.694
		(0.152)		(472; 686)	(0.070)		(556; 841)	(0.126)		(443; 575)
	Bad	0.222	[-0.256, 0.701]	2.804	0.281	[-0.117, 0.680]	3.101	0.053	[-0.325, 0.432]	3.071
		(0.244)		(207; 327)	(0.202)		(214; 379)	(0.193)		(213; 363)
Chronic diseases	No chronic diseases	0.251*	[-0.030, 0.532]	3.481	0.002	[-0.126, 0.131]	4.279	-0.029	[-0.365, 0.306]	2.295
		(0.143)		(442; 683)	(0.066)		(558; 824)	(0.171)		(333; 418)
	Otherwise	0.162	[-0.175, 0.499]	3.097	0.180	[-0.079, 0.440]	3.593	0.156	[-0.085, 0.397]	3.960
		(0.172)		(285; 480)	(0.132)		(324; 557)	(0.123)		(359; 611)
Income (net of pension)	Low	0.488**	[0.038, 0.937]	2.632	0.270*	[-0.143, 0.345]	3.029	0.270*	[-0.090, 0.645]	3.029
		(0.229)		(667; 928)	(0.152)		(472; 686)	(0.152)		(472; 686)
	High	-0.013	[-0.225, 0.198]	4.537	0.077	[-0.098, 0.253]	4.062	-0.026	[-0.175, 0.122]	4.629
		(0.108)		(514; 757)	(0.089)		(424; 696)	(0.076)		(528; 764)

Robust criteria errors for clustering to the individual level are reported in parentheses, *p < 0.1, **p < 0.05

Table 3 also shows that self-rated moderate or healthy individuals had a significant 27 percentage point increase in the probability of discretionary OTC drug purchases in response to the NPRS pension shock. The income shock effect on the group that answered poor health was not significant. Similar results were found for the subsample on depression and chronic diseases, where the effect of NPRS on the utilization of health services, including over-the-counter drugs, were not significant, but on the receipt of NPRS, the probability of discretionary OTC drug purchasing behavior increased among rural residents who did not suffer from depression (28%) and did not have chronic diseases (25%). We believe that individuals in poor health, experiencing depression, and self-rating their health as unhealthy might have increased prescribed drug purchases as part of curative inpatient and outpatient services. In contrast, discretionary OTC drug purchases were mainly for symptom relief and disease prevention [41]. The receipt of NRPS relaxed the budget constraint on individual consumption behavior that provided funds for discretionary OTC drug purchases, especially among those who were without chronic diseases and knowledgeable about their health.

Finally, we divided the sample into low- and high-income groups using the mean value of income. The results in Table 3 show that receipt of NRPS mainly influenced the over-the-counter drug purchases of the low-income group, with the probability of discretionary OTC drug purchases by the low-income group increasing by about 50 percentage points. This is consistent with our theoretical framework where income reflects the ability to access related resources and is an important factor influencing the utilization of discretionary drug services. The budget constraint is weaker for the high-income group, while the demand for discretionary drugs is suppressed for the low-income group before receipt of the NRPS. The pension income brought by the NPRS relaxed the budget constraint for the low-income group.

### Robustness tests

To test the robustness that over-the-counter drug utilization was driven by the pension, we conducted continuity, data heaping, placebo and regressions using different bandwidths and cutoff tests.

- 1. Continuity test

**Fig. 3:**
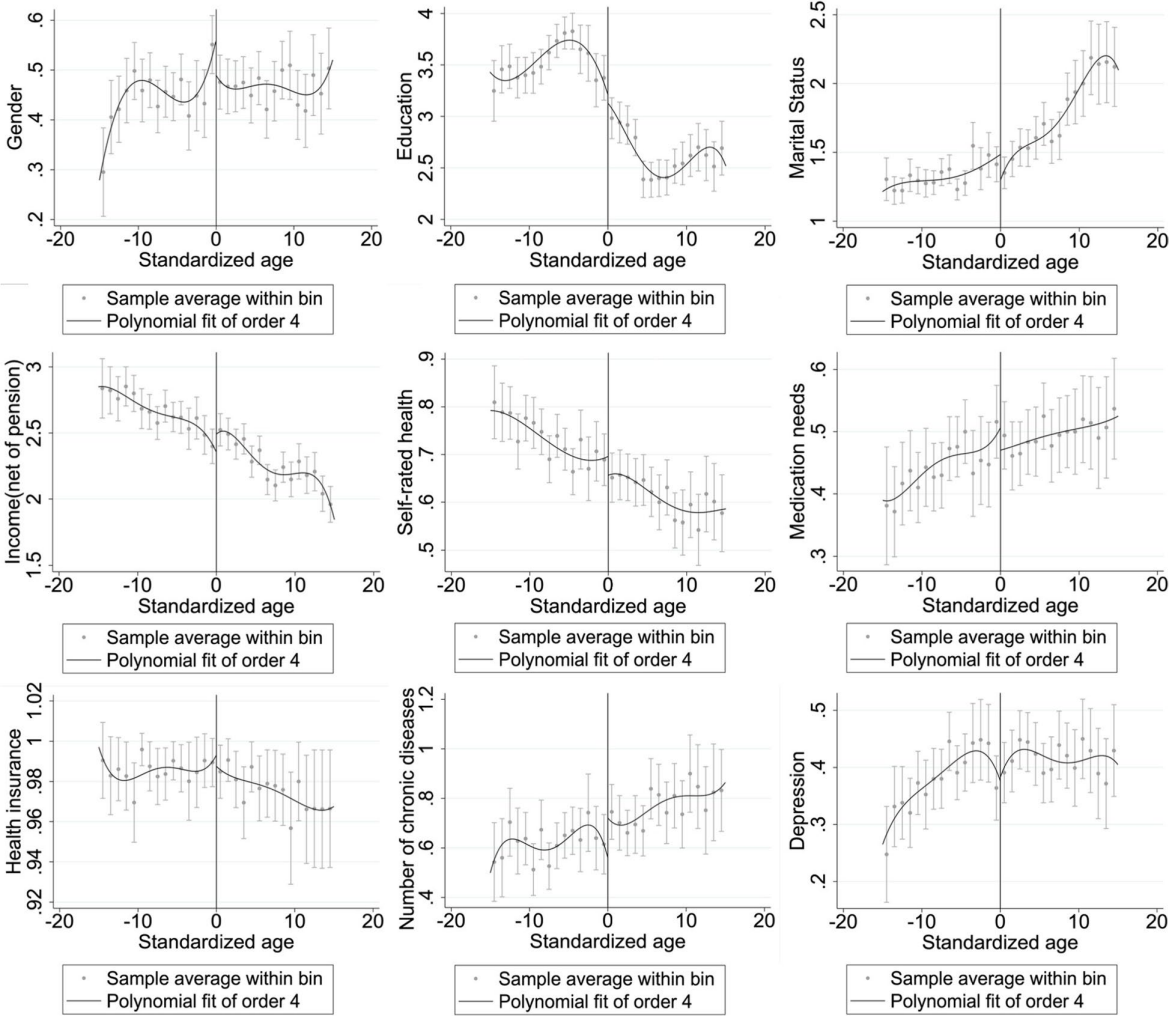
Continuity test

The prerequisite of applying discontinuity regression is that all predetermined variables are free from discontinuity at the cutoff. All covariates were tested by discontinuity regression, and the regression results are shown in Table 4 and Fig. 3. We first tested the continuity of each predetermined variable at the cutoff, and the regression settings are the same as in the previous regressions, except that the dependent variables are replaced with covariates. In Table 4, the estimation results for variables (1)-(9) show that there is no discontinuity at the cutoff for all the antecedent variables.

- 2. Data heaping test

**Table 4:** Continuity test

		Pension	95% CI	Bandwidth	Sample size
(1)	Health insurance	-0.03014	[-0.118,0.058]	2.312	556; 763
		(0.04525)			
(2)	Depression	0.00078	[-1.994,0.195]	3.649	781; 1261
		(0.09942)			
(3)	Education	-0.61758	[-1.950,0.715]	2.251	556; 763
		(.68006)			
(4)	Marital Status	-0.36904	[-0.918,0.180]	3.248	718; 1119
		(0.28038)			
(5)	Number of chronic diseases	0.24649	[-0.437,0.930]	2.405	581; 788
		(0.34909)			
(6)	Medication needs	-0.15099	[-0.347,0.045]	3.811	819; 1321
		(0.10045)			
(7)	Sex	-0.10119	[-0.231,0.029]	4.861	1188; 1667
		(0.0665)			
(8)	Self-rated health	-0.01528	[-0.183,0.152]	3.998	862; 1379
		(0.08584)			
(9)	Income (net of pension)	0.42971	[-0.196,1.056]	2.804	655; 941
		(0.31961)

**Fig. 4:**
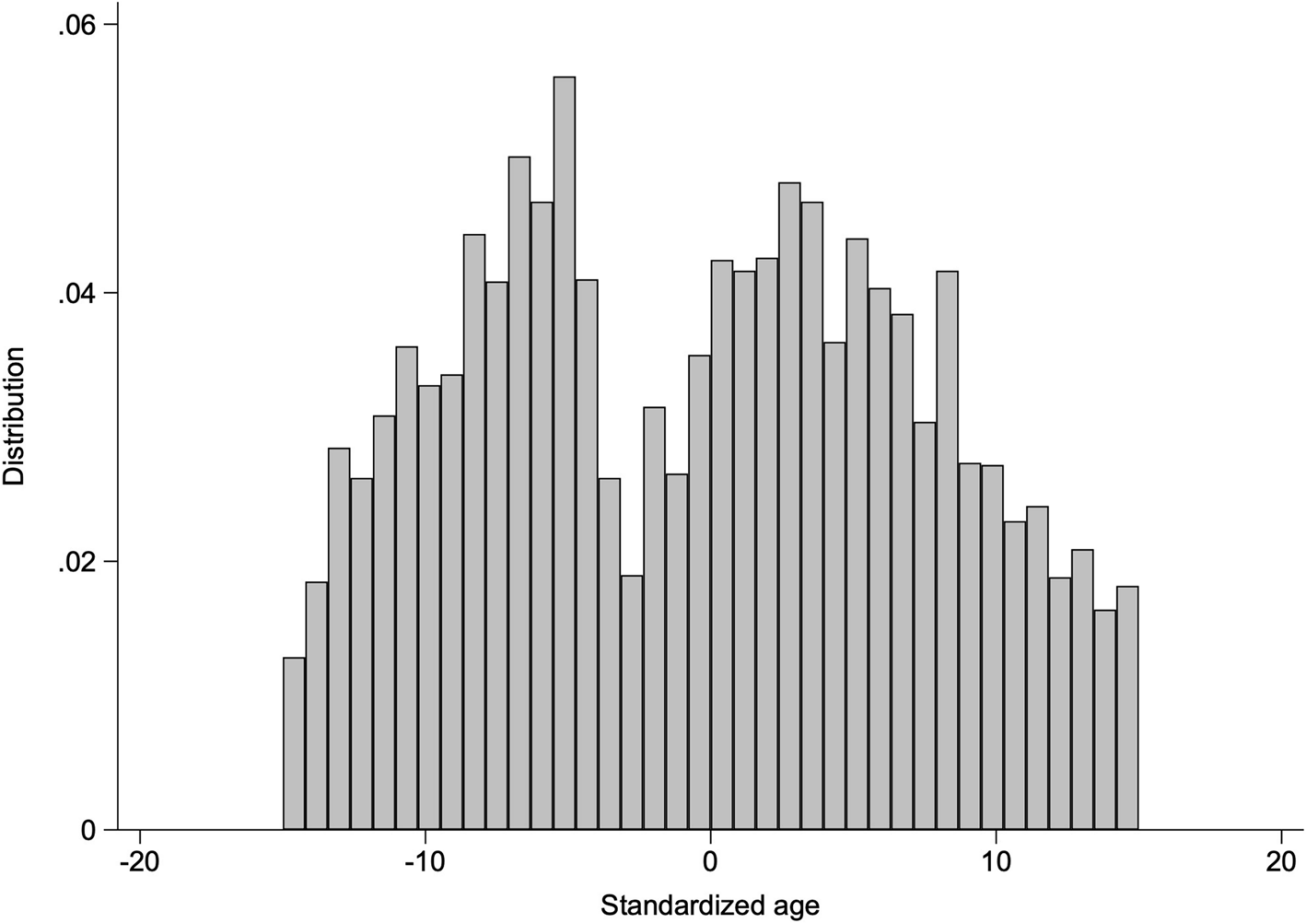
Histogram of age distribution

The fulfillment of the continuity assumption requires that there is no data heaping in the sample population at the cutoff [42]. To test for data heaping, a histogram of the data distribution is shown in Fig. 4, and it is clear from Fig. 4 that there is no data heaping.

- 3. Placebo test

The placebo test shows that the health care burden is affected only by the pension income shock, and not by other factors, when the regression results are insignificant at other ages away from 60. The regression results should be insignificant when we make a "pseudo cutoff" at other ages away from 60 years old. In Table 5, the regression results for "pseudo-cutoff" ages 57, 58, 59, 61, 62, and 63 were not significant, which suggests that the increase in the probability of discretionary OTC drug purchase near the age of 60 was due to NRPS.

**Table 5:** Placebo test

	(1)	(2)	(3)	(4)	(5)	(6)
Cutoff	57	58	59	61	62	63
	Probability of discretionary OTC drug purchase					
Pension	-0.36698	0.69171	0.87341	0.32878	0.28203	-2.1045
	(0.6513)	(0.60854)	(1.112)	(0.65295)	(0.28669)	(6.7784)
95% CI	[-1.643,0.909]	[-0.501,1.884]	[-1.305,3.052]	[-0.950,1.608]	[-0.279,0.843]	[-15.389,11.180]
Bandwidth	3.233	2.614	2.096	3.038	3.119	2.930
Sample size	1047;768	586;700	416;666	821;1081	953;1090	994;1015
	Inpatient					
Pension	-1.0819	-0.01068	0.0613	0.24706	0.06453	-0.30709
	(0.66936)	(0.23101)	(0.66866)	(0.29373)	(0.22644)	(0.57976)
95% CI	[-2.393,0.229]	[-0.463,0.442]	[-1.249,1.371]	[-0.328,0.822]	[-0.379,0.508]	[-1.443,0.829]
Bandwidth	3.079	4.188	2.143	5.188	3.096	3.687
Sample size	985;710	1241;1226	416;666	1266;1777	953;1090	1217;1236
	Outpatient					
Pension	-1.7666	0.35675	0.27627	1.1342	-0.06784	-0.04335
	(1.954)	(0.30082)	(0.64699)	(1.4924)	(0.22001)	(0.32914)
95% CI	[-5.596,2.063]	[-0.232,0.946]	[-0.991,1.544]	[-1.790,4.059]	[-0.499,0.363]	[-0.688,0.601]
Bandwidth	2.551	3.191	1.544	3.542	3.013	4.035
Sample size	781;565	827;907	333;469	946;1223	934;1065	1302;1333

- 4. Regression using different bandwidths

Our optimal bandwidth for the baseline regression was symmetric about both sides of the cutoff. Different bandwidths on both sides of the cutoff were applied as a robustness check [37]. Table 6 shows that the regression results were still significant with a bandwidth of 2.861 years on the left side of the cutoff and a bandwidth of 2.380 years on the right side. We also customize 4 years as the bandwidth, and the results remain robust.

- 5. Cutoff

**Table 6:** Estimation results at different bandwidths

	Panel A		Panel B		Panel C	
	Discretionary OTC drug purchase		Inpatient		Outpatient	
Pension	0.28673*	0.17292*	0.10006	0.07948	0.11012	0.09868
	(0.14995)	(0.09106)	(0.08782)	(0.06601)	(0.10165)	(0.06991)
95% CI	[-0.007,0.580]	[-0.005,0.351]	[-0.072,0.272]	[-0.049,0.208]	[-0.089,0.303]	[-0.038,0.235]
Bandwidth	2.861; 2.380	4	2.688; 3.904	4	2.867; 2.930	4
Observations	663;788	862;1379	645;1349	862;1379	663;1019	862;1379

Robust criteria errors for clustering to the individual level are reported in parentheses, *p < 0.1, **p < 0.05, ***p < 0.01

For rural residents, there is no other policy that uses age 60 as a decomposition [43], so the effect of the age 60 cutoff can be attributed to the NRPS income shock.

## Discussion

Our results show that the NRPS income shock had no significant effect on inpatient and outpatient utilization among rural residents, but significantly increased the probability of discretionary OTC drug purchases. We offer possible explanations. First, outpatient and inpatient services are unpredictable and irregular, and both outpatient and inpatient hospital services are costly one-time expenditures. An RMB88 (USD12.97) monthly pension income shock cannot address large inpatient and outpatient expenses. Second, different from inpatient and outpatient hospital services, the cost of discretionary OTC drug purchases was small. Whether short-term for a specific illness or regular preventative therapies, it is well-suited for payment by predictable and regular income supplements brought by NRPS. Evidence suggests that additional non-labor income shocks can increase and improve health status and discretionary OTC drug purchasing was a potential pathway for regular non-labor income shock spending [44–46]. Third, the price elasticity of demand is more elastic for drugs than for outpatient and inpatient services [47, 48]. Our finding suggests that the relaxation of income constraints after receiving a pension can explain a significant increase in the probability of discretionary OTC drug purchasing, but not for inpatient and outpatient visits. Fourth, in terms of social security payments, small income shocks may have a significant impact on drug purchasing in countries with less well-developed social security systems [49–52]. Literature focused on the effect of NRPS on alleviating the health care spending burden argued that subsidize income is better than subsidize health insurance [53].

Our result is consistent with the former research focus on pensions in rural China, which also found that pensions encouraged rural people to choose self-treatment [16]. They found that pension did encourage people in the lowest 25% income group to use both outpatient and inpatient services [16]. When we focus on the lowest income quartile, we expect similar results. However, the sample size after dividing our sample into four equal parts was not sufficient to perform RDD. There is also a literature that finds that NRPS can alleviate the healthcare burden of rural residents [53]. For other low- and middle-income countries (LMICs), a literature review suggests that cash transfers may increase healthcare expenditure [54].

We also found that the increased probability of discretionary OTC drug purchases was not due to age-related deterioration in health. Table 4 and Fig. 3 show that self-rated health, number of chronic illnesses, and depression did not change significantly around the cutoff.

Our data suggest that rural residents faced adverse health conditions and drug needs before the NRPS 60-year-old age. Pre-NRPS, rural residents’ low-income levels meant the absence of financial resources to obtain healthcare services [55]. The subsample analysis reconfirmed this proposition. There was no significant change in the health status of the rural elderly before and after receiving the pension. The impact of the NPRS was concentrated on people with better health and lower income. These people had preventive medical needs, but their need for discretionary OTC drug purchases was suppressed pre-NRPS due to budget constraints. The exogenous NPRS income shock helped release the suppressed demand for discretionary drugs, especially by those with an awareness of their health needs.

The health effects of over-the-counter drugs are well documented. As more and more drugs become available over-the-counter, discretionary OTC drug purchases, which are usually available in pharmacies, offer people the opportunity to promote their own health [56]. First, self-medication with over-the-counter drugs is increasingly becoming the choice of treatment for common self-limiting conditions. If over-the-counter drugs are widely available and have a low abuse rate [57], they can address common symptoms, such as fever and pain relief, and health recovery [25, 56, 58, 59]. Second, over-the-counter drugs enable patients to cultivate their awareness and behavior of self-managed healthcare [27, 28, 60]. Self-managed over-the-counter drug purchases are conducive to relieving disease symptoms and reducing disease harm effects before more severe disease happens [61].

Our data do not allow us to further identify the type of over-the-counter purchased by respondents. A national audit released by IQVIA (2022) shows that in 2021, over-the-counter drugs accounted for 42% of the overall retail pharmacy market in terms of sales share [62]. Traditional Chinese medicine (TCM) accounted for 52% of the over-the-counter drug healthcare market, with TCM more common for preventive healthcare than for curative care [24].

We make recommendations for the health promotion of rural residents to attenuate the national public health burden. Prior to receiving their pensions, rural residents experienced ongoing illnesses, but budget constraints suppressed their consumption of discretionary drugs. Delaying medication treatment accelerates disease progression and reduces patient quality of life [63]. From a public health and national burden of disease perspective, suppression of healthcare needs often leads to disease exacerbation, causes augmented disease risk and constraints disease treatment and prevention, which increases the social burden of long-run healthcare [64, 65]. In addition, in the individual perspective, studies suggested that the elderly who adopt self-medication are less likely to use inpatient services, and their hospitalization costs are significantly lower [21]. The economic value of self-medication among the Chinese elderly population is also significant at the individual level.

While recognizing China’s public health budget constraints, we recommend expanding access to healthcare services to the rural population to promote optimal self-medication drug use. Self-medication behaviors with over-the-counter drugs were prevalent in China, which was related to residents’ socio-demographic characteristics and health literacy [17]. For rural populations, we recommend including commonly used over-the-counter drugs in basic health insurance reimbursements to enhance drug accessibility. To ensure optimal self-administered over-the-counter drugs, the government should run an awareness campaign on preventative health, especially focusing on the rural poor. Inappropriate use of over-the-counter drugs can also lead to deterioration of health status and a corresponding financial burden when patients lack knowledge about the side effects of drugs and their usage regimens [66, 67]. Over-the-counter drug use should ideally be part of professional advice by doctors and nurses in local rural health facilities, forming part of a preventative healthcare check. There is evidence that self-administer over-the-counter drug purchases can provide doctors with information about undiagnosed diseases [26, 61]. We recommend that during regular medical check-ups in local health facilities, doctors ask patients about their over-the-counter drug purchases to help identify potential health problems.

China’s rural pension scheme provides a practical example of improving social and healthcare security in other developing countries. One important lesson for other countries from the NRPS impact on over-the-counter drug utilization is that relatively small monthly income supplements can impact discretionary OTC drug purchasing behavior, with potential short and long-run health outcomes.

The paper has a number of limitations. First, our data do not allow us to further identify whether the increased probability of discretionary OTC drug purchases is an effective demand release or overuse of OTC drugs. Second, our results are limited to rural residents. There exists heterogeneity in medical behavior between rural residents and urban residents, so further studies are required to determine income shocks on urban residents’ drug purchasing. Third, our research only focuses on the NRPS’ impact on over-the-counter drugs, purchasing behavior for prescription drugs requires further empirical testing.

## Conclusion

Stepping outside the framework of previous work on the impact of health insurance on medical service utilization, we explore the impact of China’s rural pension scheme on rural residents’ healthcare utilization. We found that the NRPS pension, as an income shock, significantly increased the probability of discretionary OTC drug purchases, but had no significant effect on the utilization of outpatient and inpatient services. Discretionary OTC drug purchase was more pronounced among healthier rural residents and those with lower incomes. Various robustness tests confirmed our results. Income shocks might relax budget constraints and time limits to promote self-medicated over-the-counter drug purchases. We suggest that the Government and doctors should pay more attention to self-medicating behaviors such as the usage of over-the-counter drugs in rural areas. Our research findings hold significant implications for policymakers and researchers, as they can serve as a basis for formulating and implementing targeted interventions to optimize the allocation and utilization of healthcare resources within rural communities.

## Data Availability

The datasets analysed during the current study are available in the China Health and Retirement Longitudinal Study (CHARLS) repository, [http://charls.pku.edu.cn] [39].

## Abbreviations

NRPS: New Rural Pension Scheme
FRDD: Fuzzy regression discontinuity design
CHARLS: China Health and Retirement Longitudinal Study
CHNS: China Health and Nutrition Survey
ORPS: Old Rural Pension Scheme
TCM: Traditional Chinese medicine
